# The rise of bioelectronic medicine

**DOI:** 10.1186/s42234-024-00151-8

**Published:** 2024-08-21

**Authors:** Dimitrios A. Koutsouras, George G. Malliaras, Geert Langereis

**Affiliations:** 1 IMEC NL, 5656 AE Eindhoven, The Netherlands; 2 Electrical Engineering Division, Department of Engineering, University of Cambridge, 9 JJ Thomson Avenue, Cambridge, CB3 0FA UK

**Keywords:** Bioelectronic medicine, Implantable bioelectronics, Medical devices, Neuromodulation, Closed-loop and targeted therapies

## Abstract

Bioelectronic Medicine (BEM), which uses implantable electronic medical devices to interface with electrically active tissues, aspires to revolutionize the way we understand and fight disease. By leveraging knowledge from microelectronics, materials science, information technology, neuroscience and medicine, BEM promises to offer novel solutions that address unmet clinical needs and change the concept of therapeutics. This perspective communicates our vision for the future of BEM and presents the necessary steps that need to be taken and the challenges that need to be faced before this new technology can flourish.

## The promise of BEM

In modern medicine,drugs produce their therapeutic effects by interacting with specific biological targets. However, as drugs are released in the blood stream, they also interact with healthy tissue, often for extended periods of time. As drug specificity is not perfect, this leads to toxicity and off-target effects (Olofsson et al. 2017). Although localized drug delivery minimizes these side effects by releasing the drug only in the proximity of its target, drug administration in general is usually not optimized to the needs of individual patients. This results in lower or higher doses than the optimal ones and leads to poor therapeutic outcomes.

Bioelectronic Medicine (BEM) treats disease by stimulating electrically active tissues. Implants are surgically placed inside the body and stimulate sites in the brain, spinal cord, peripheral nerves, but also the heart and various muscles. The effect can be local, or not: As the nervous system innervates every organ in the human body, an implant can selectively target and modulate the activity of an organ (e.g. spleen) through the nerve that innervates it. In this context, disease symptoms can be effectively treated by electrical signals delivered inside the body by miniaturized bioelectronic devices that act on the nervous system, replacing pharmacotherapies.

BEM promises to treat a variety of health conditions with reduced side effects and improved efficacy compared to drug administration (Famm et al. 2013; Zhirnov 2018). The use of microelectronics and information technology paves the way to the delivery of personalized, targeted, and on-demand treatments. For example, physicians program the implant to deliver to their patient the appropriate stimulation “dose” that maximizes benefits while minimizing side effects. In the future, we can envision dose adjustments that are implemented by a physician who remotely programs the implant based on updated patient information. In a paradigm changing scenario, the implant can be turned on only when needed, or its stimulation be adjusted through more sophisticated “closed-loop” approaches that capture and analyze bioelectronic signals of, e.g., an oncoming epileptic seizure, in real time.

In addition to safety and efficacy advantages, BEM allows for better control of the environmental impact of the delivered treatment compared to pharmacological solutions. Materials from the implant could be potentially recovered and recycled. Thus, unlike pharma-based therapies, there could be reduced materials ending up in the surface water. BEM solutions, and specifically the ones utilizing battery-less devices, promise a patient life cycle with reduced need for hospitalization and expert consulting, leading to cost-efficiencies. Once implanted, BEM devices could save a lifetime of drug administration thanks to the implant lower maintenance need and cost compared to the pharmacotherapy cost especially for chronic diseases (Sarica 2021).

Bioelectronic medicine goes back to classic times, with repots of electric fish being used to treat migraines (Tsoucalas et al. 2014). The famous experiments of Luigi Galvani in the eighteenth century, where the legs of a dead frog were made to twitch after application of electricity, helped bring bioelectricity to the forefront (Loeb 2005). Two centuries later, in 1958, the first fully implantable pacemaker for treating cardiac arrythmias was developed (Jeffrey and Parsonnet 1998). In 1961, the first cochlear implant to treat profound deafness was presented (Mudry et al. 2013), and from that point on the scientific community started to realize the enormous potential of bioelectronic medicine. Since then, BEM has moved in multiple directions (Sdrulla et al. 2018; Koopman et al. 2016; Mayberg et al. 2005; Benabid et al. 1996; Clavo et al. 2012; Milby et al. 2009). Currently, the markets of Spinal Cord Stimulation (SCS) with a value of $ 2.92 billion (2023) (Expert Market Report 2024), Deep Brain Stimulation (DBS) with a value of $1.41 billion (2023) (The Business Research Company 2024), (The 2014, and Vagus Nerve Stimulation (VNS) with a value of $479.15 million (2023) (Reports VM 2023), are the main ones to which BEM finds direct application for indications that include chronic pain, Parkinson’s disease and other movement disorders, and drug-resistant epilepsy. However, much effort is being made to apply bioelectronic medicine to many other conditions, including cardiovascular, autoimmune, and metabolic diseases. The field is promising but still in its infancy, requiring many milestones to be reached before it fulfils its true potential. These milestones are either technological, namely advances in the fabrication of microscale bioelectronic devices able to bilaterally interact with biological tissue at cellular level, or biological, namely a deeper understanding of anatomy, physiology, and the mechanisms underlying human pathology. Provided that these milestones will be achieved, BEM can be a complementary therapeutic approach to pharmacotherapies especially for drug resistant or difficult to treat pathologies.

## Future BEM systems

**Fig. 1:**
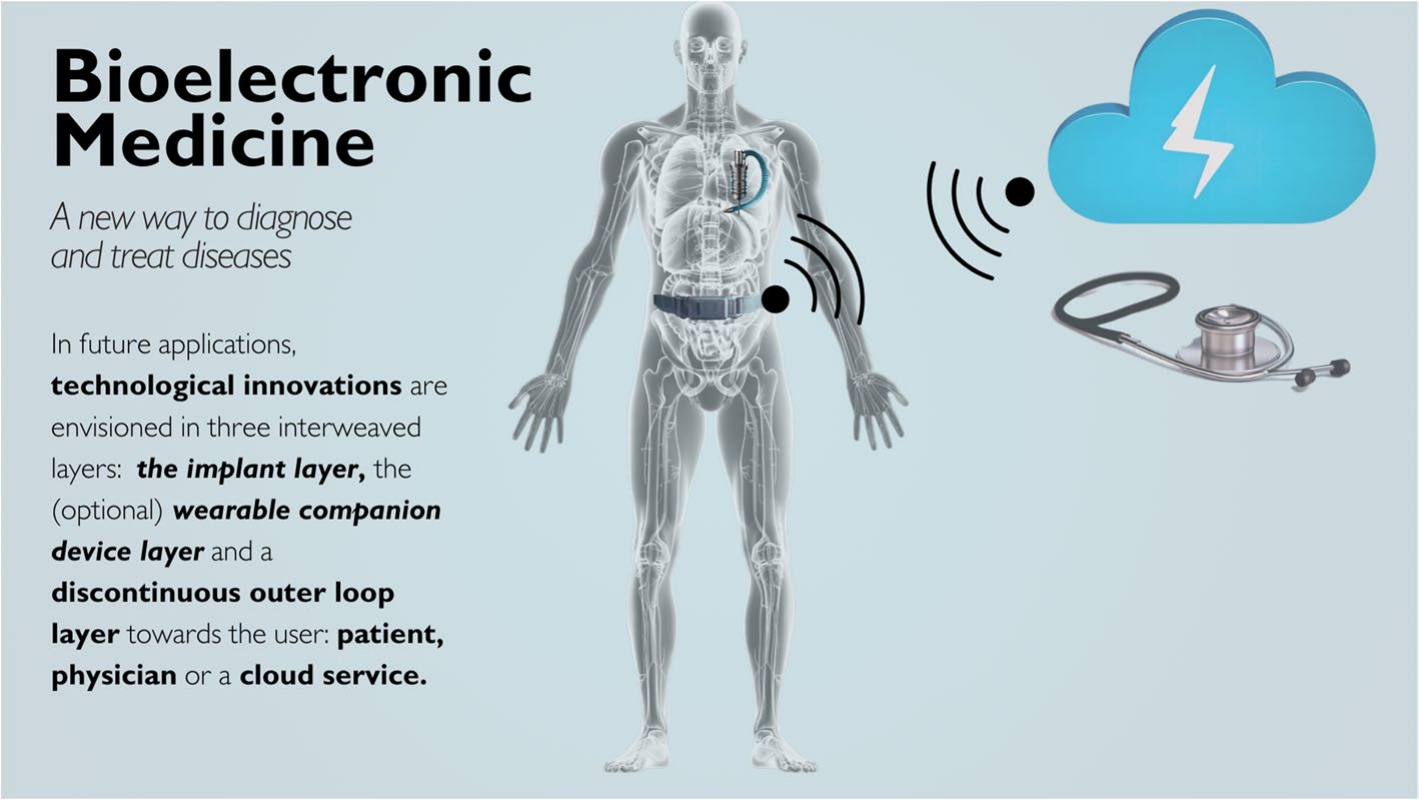
Bioelectronic Medicine (BEM) is not necessarily restricted to a single, isolated and autonomous implant. Depending on the addressed condition, there may be a wearable companion for power transfer and data exchange. Distributed BEM employs secondary implants or sensing wearables. The wearables can act as a hub to a handheld device that provides feedback to the patient and allows a certain degree of control. Exchange with the cloud allows data access to a physician or a cloud service center. A clinician controller allows the physician to adjust the delivered treatment. Data processing may take place at different nodes, depending on their computational power and energy needs

Various technological challenges must be overcome before BEM becomes a generic therapeutic approach that can be deployed to patients at scale. Here, we discuss these in a typical architecture of a future BEM system, that is distributed over three interweaved layers, as depicted in Fig. 1. The system consists of the implant layer, an optional wearable companion layer, and the user interface layer, which is an outer loop layer, used to communicate with the patient, the physician, or a cloud service.

### The implant layer

The front end of the implant layer is typically an electrode array that stimulates the tissue. This is the interfacing point between human technology and biology. Some implants are already bidirectional, i.e., in addition to stimulating tissues, they are also capable of recording. The recording capabilities are used for capturing biological signals from the tissue. In future implants, the concept of bidirectionality will be generalized by, for example, an *actuator loop* which will be used to evaluate the effectiveness of stimulation on the tissue. This loop will be able to automatically adjust the stimulator if necessary, and at low latency, to ensure the timely delivery of the bioelectronic medicine therapeutic doses. Future implants will also become multimodal and integrate capabilities including biosensing and drug delivery.

In most current implants, the front end is connected to an implantable pulse generator (IPG) that contains the battery and electronics in a hermetic package that protects these components from the bodily fluids, guaranteeing biostability (Sarica 2021; Denison and Morrell 2022). The connection between the “wet” front end and the “dry” electronics in the IPG is a significant challenge. Chronic implants require the packaging and encapsulation to be functional for at least 5–10 years, highlighting the magnitude of the challenge. As implants become increasingly miniaturized, bidirectional and multimodal, the challenges posed on the electronics in the IPG increase dramatically. For example, multiplexing becomes necessary for implants with large electrode counts (Drakopoulou et al. 2023). A solution that is increasingly being adopted in research involves merging the electronics and power with the front end. This, in turn, requires new encapsulation methods that ensure hermeticity of electronics while maintaining compatibility with flexible or even stretchable form factors. At the same time, it drives changes in the design of the implant’s components.

One such change, dictated by current trends, involves battery-less implants. Wireless powering techniques can be based on several energy transfer methods, although inductive/electrical (Habibagahi 2022; Bocan et al. 2016) and ultrasound (Taalla et al. 2019) are the most studied ones. Other methods such as magneto-electric (Chen et al. 2022) or optical (Doguet et al. 2024) power transfer are also reported. Battery-less implants will be easier to miniaturize, avoid the need for battery changes, and have improved environmental footprint (Sarica 2021). Regardless of whether the battery is small, or even absent, the low power design of the architecture and microelectronics remains always a technical challenge.

Additional changes in implant design are imposed by the need to transfer data in and out of the body. The choice of the communication system is a matter of full system partitioning where the trade-off between local pre-processing and transmission of data is a design parameter (Hueber 2023; He et al. 2022) In some cases multiple implants are envisioned as part of a therapy, in a therapeutic mode that may require implant to implant communication and synchronization (Bereuter 2018). This calls for circuitries that need to address multiple channels and for on board processing functionalities. Neuromorphic circuits can offer their power efficient information processing properties for the realization of edge computing paradigms at the implant level.

Finally, the choice of materials in future implants will be expanded. At the level of electrodes, material science research offers alternatives to the typically used metals (Cogan 2008). For example, conducting polymers have recently emerged as candidates to seamlessly bridge the gap between the worlds of biology and electronics due to their unique properties which include mixed ionic/electronic conductivity, mechanical flexibility, and enhanced biocompatibility (Dong 2013). They offer reduced impedance compared to traditionally used electrodes, which allows miniaturization that promotes treatment selectivity and reduction of off-target effects (Cui and Martin 2003; Koutsouras 2017). At the same time, they reduce the voltage overshot during stimulation, improving thus safety and decreasing energy consumption (Wilks et al. 2019). In other approaches, graphene (Kuzum et al. 2014), MXenes (Driscoll et al. 2015), or carbon nanotubes (Vitale et al. 2015) have been already successfully used in bioelectronic medicine applications due to their novel electronic and optical properties.

Additionally, all materials that come in contact with the body should minimize any adverse effects on it, ensuring biocompatibility. Foreign Body Reaction (FBR) is a process that inevitably occurs whenever a foreign material is inserted into the body (Carnicer-Lombarte et al. 2021). The implantation, traumatizes the surrounding tissue and triggers a cascade of events known as inflammatory reaction. To minimize the FBR, there is currently a trend in shifting from the traditional stiff and rigid electrodes to more flexible, stretchable and soft ones which bridge the mechanical mismatch with the biological tissue (Someya et al. 2016). Going a step further the idea of a “living electrode” has been introduced lately to minimize the FBR (Goding et al. 2018). In this approach, a cell layer is integrated into the bioelectronic device and acts a functional layer minimizing the biotic/abiotic mismatch. Especially in the field of neuroprosthetics, novel biohybrid neural interfaces can establish a chronically stable, quick quality communication with e.g. peripheral nerves (Rochford 2023).

### The wearable companion layer

In some cases (e.g. cochlear implants), a wearable companion is used to wirelessly transfer power and data to the implant (Macherey and Carlyon 2014). Although the concept of a wearable companion is not practical for a lifesaving implant such as a pacemaker, it can be envisioned for the more subtle treatment of chronic diseases. The choice of the communication protocol from a wearable companion to the implant depends not only on the required data rate, but on many system specifications and user scenarios. The most common medical implant communication systems (MICS) are based on the 401–406 MHz band (Singer et al. 2021; Islam et al. 2016), but Bluetooth and low-bitrate modulation on top of the powering system are also foreseen.

A wearable companion device also enables measurements on the outside of the body (Dunn et al. 2018). In future chipsets for implants there must be inputs to close therapeutic loops and make them adaptive, and the wearable companion can perform measurements that provide such inputs. Additionally, the wearable companion may run an *adaptive therapy loop*: an adaptive system that detects the therapy effectiveness by monitoring the effect on the targeted end-organs or systemic physiological functions. The goal is to titrate the therapy by adjusting the stimulation intensity. This loop is dependent on the measurement of a specific organ or a systemic function biomarker (for example heart rate, glucose level or inflammatory response), and can have a latency of minutes or even hours.

### The user interface layer

Besides an *actuator loop* on the implant, and an *adaptive therapy loop*, potentially in conjunction with a wearable, there will always be a discontinuous *chronic therapy loop* where the physician adjusts the therapy in a consult-based fashion. Sometimes, the user can also change some parameters within certain boundaries to increase the perception of self-control. This outer loop can be assisted by a digital twin (Erol 2020) or a decision support system.

With the use of a (partially) implanted closed-loop system, the data processing will be distributed over the three layers to optimize for transmission data-rate, computational power, and electronic power making the therapy adaptive. It is already known from control theory that closed-loop systems are more stable and can withstand temporary disturbances. Closed-loop systems for BEM are predicted in an early phase (Famm et al. 2013) and referred to in the IEC60601-1 regulations (Zhirnov 2018). The therapeutic advantages are known in the medical field (Zanos and Zanos 2019), and already deployed in commercial products (The Evoke® System | Saluda Medical 2023).

All three aforementioned loops optimize the efficacy of the therapy, but with fundamentally different time constants, latency requirements, level of physiological sensing, and forgiveness in their judgement error.

## The way forward

The bioelectronic medicine field is innovative and exciting as it paves pathways to novel therapeutic paradigms which satisfy unmet medical needs. Nevertheless, people should be cautious about the hype that might be created and the risk of developing unrealistic expectations of the technology. There are still challenges that need to be faced before future bioelectronic medicine systems make it to the clinic at scale. These challenges go beyond simple hardware issues and include a better understanding of the targeted biological mechanisms, and the need to develop suitable translational ecosystems that address regulatory and ethical issues, and health economics.

A better knowledge of human anatomy and physiology is essential in realizing improved therapeutic approaches. A precise mapping of neuronal networks and a deeper understanding of the complex interplay between the nervous system and the innervated organs can unlock novel therapies. This can be achieved by the identification and classification of the neuronal activity patterns that govern the functioning of the nervous system. The encoded information in these patterns can shed light on the way neuronal networks communicate, allowing neuroscientists to answer fundamental scientific questions. In parallel, neuropathological activity can be used as a disease biomarker. For example, electrocorticographic activity can be used as a marker for the detection of epileptic seizures, in a treatment mode which is agnostic to the inaccuracy that accompanies the patient self-reporting process (Arcot Desai et al. 2019). The pallet of biomarkers can be extended to the use of biochemical markers. For example, Interleukin 6 (IL-6) can be used as an inflammatory marker (Rincon 2019). Regardless of the nature of the biomarker, the information that accompanies its acquisition will be processed and interpreted to become input to a closed-loop system that can deliver a personalized, efficient, and targeted therapy (Famm et al. 2013).

The use of devices that interface with the nervous system also raises concerns regarding data security, identity, agency, and autonomy of the patient. Since bioelectronic medicine systems are expected to collect and store biometric data, care should be taken to guarantee that this information is not leaked in order to ensure patient privacy. Issues also exists regarding the potential of the technology to affect thoughts, emotions, or memories whistleblowing for the imperative need of safeguards to protect patients from losing their identity or the control of their decisions and actions. Even if we assume the use of neurotechnology purely as a means of enhancing natural human abilities, concerns are still raised about the potential violation of the social justice and equality, for example during a job interview process where applicants with artificially enhanced abilities will be competing against ones with no artificial implants. Abandonment of neurotechnology is also currently in the midst of a lively debate. Many are raising red flags about the risk to patients receiving neurological implants if neurotech companies decide to discontinue their products and stop providing the necessary software updates to keep them effective. Steps should be taken to mitigate this risk by ensuring that companies can guarantee lifelong technical support for patients who decide to trust their bodies to neurotechnology.

Cost effective fabrication and mass production of bioelectronic medicine devices is still an open research topic. New electrode form factors and new materials are needed for more targeted and efficient stimulation paradigms with reduced off-side effects. Nevertheless, a change to the way BEM devices are fabricated is also imperative. Currently, many neurotechnology companies in the world still produce devices manually under a microscope. The way forward dictates leveraging the infrastructure of modern microelectronic industry where fabrication is fully automated. Therefore, electrodes down to the microscale level can be produced with exceptional precision, high throughput and reproducibility, features of immense importance to bioelectronic medicine. In the long term, multiple implant constellations are envisioned which will add to the complexity of the fabrication process since each one of them will have to accommodate different tissue topographies. Nevertheless, the business opportunities lie in the creation of production lines that leverage the accumulated knowledge of the already established high-tech industries and the repositioning of successful fabrication approaches from other fields in the service of the bioelectronic medicine technology.

Future bioelectronic medicine systems will not only consist of a single autonomous implant but will potentially comprise multiple implants, wearable companions and connections to hand-held devices and the cloud. The implants will make use of modern architectures where microcontrollers running firmware will be updated without the need for hardware replacement. Although software and firmware flexibility and network connectivity present enormous opportunities regarding the upgrading of the already installed product base, they come with a new service business model as well, since the healthcare ecosystem and technology servicing is not yet used to large software component in the medical devices.

The placement of current state-of-the-art implants requires invasive surgical procedures and therefore, BEM-based treatments come with a high upfront cost compared to drug-based treatments. With new miniaturized BEM devices, however, implants will become less invasive, changing the economic model. This will also enable BEM-based treatments to be prescribed for the treatment of an expanding array of conditions, including those associated with less severe symptoms than the ones treated today.

Similarly to any new drug that is introduced to the market, bioelectronic medicine devices will need to undergo a study before they get approval for safety and effectiveness. In the USA the approval is given by the Center for Devices and Radiological Health (CDRH) which belongs to the Food and Drug Administration (FDA) agency. In Europe, the European Medical Device Regulation (EMDR) provides a set of regulations which controls the fabrication and circulation of any new medical device. The trials usually involve fewer participants and longer follow ups compared to drug studies realizing a more lifecycle-oriented approach to medical device regulation (Denison and Morrell 2022). One of the main challenges which bioelectronic medicine devices are expected to face is the fast-paced changes in the hardware and software technology (and the increasing use of artificial intelligence) that propels progress in the field but also increases the demand for constantly up to date regulatory schemes to follow these advancements. Furthermore, the use of new and innovative BEM platforms (e.g., biohybrid devices) is muddying the landscape by posing additional challenges in establishing well-defined regulatory frameworks for safe and effective use. Therefore, the need for new standards to cover this novel technology is imperative.

Regarding the implementation of bioelectronic medicine in clinical practice there are also disadvantages of the technology that need to be taken into account. BEM is not entirely free of side effects. VNS is linked to voice hoarseness, DBS of the subthalamic nucleus (STN) is considered to impact the patients’ social adjustment and their relationships in their family and professional environment, while there are surgical related adverse events for them such as infection or scarring during and after the operation procedure (Giordano et al. 2017; Schüpbach 2006). Another problem is the loss of the therapeutic efficacy due to habituation and the development of tolerance to the neurostimulation treatment which, although sometimes overlooked, is an established concept in the medical community (Fishman et al. 2019). Additionally, the long-term effects of stimulation on the human body are not, yet, well understood, raising safety concerns of the therapy for the future. On the device level, the malfunction, breakage or even migration of the implantable electrodes are issues that still have not been addressed properly while the implanted devices are not always compatible with other medical interventions, (e.g. Magnetic Resonance Imaging-MRI). There are also accessibility issues to be considered, since on the one hand the BEM treatment is not widely available to patients and on the other hand its cost is rather high. Indicatively, the DBS cost, which includes the initial consultation, pre-operative testing, the implants and other materials, the operation cost and the post-surgery care, in the US can be as high as $100.000 making the out-of-pocket payment challenging (Bishay et al. 2024). Finally, from a social point of view, patients living with implantable devices can easily be stigmatized, which causes them stress, anxiety, and feelings of shame. For example, the stigma of hearing loss leads many people to deny their hearing problems and reject the help of hearing aids (Erler and Garstecki 2002). One of the main challenges of BEM technology in the near future will be to find ways to mitigate the above problems or to prove that the benefits of using it outweigh the disadvantages of adopting it.

## Conclusions

Bioelectronic medicine aspires to offer innovative ways in disease management. By leveraging the rapid progress in fields as diverse as microelectronics, materials science, information technology, neuroscience and medicine, it aims to realize therapeutic paradigms beyond the traditional drug or surgery-based ones. The field is highly multidisciplinary, as it requires scientists with different backgrounds to work together, a fact that, by default, introduces many challenges. At the same time, though, the interdisciplinary nature of bioelectronic medicine is one of its assets as it brings together people with complementary expertise which join forces to address currently unmet medical needs. The technological and scientific challenges are many and still lie ahead, as it seems that numerous milestones need to be achieved before this technology matures. However, its enormous potential creates an exciting field for scientists and engineers to thrive. Provided there is a concerted effort from all stakeholders, the time when bioelectronic medicine will be a significant part of our standard of care may not be far off.

## Data Availability

No datasets were generated or analysed during the current study.
